# ATM-mediated co-chaperone DNAJB11 phosphorylation facilitates α-synuclein folding upon DNA double-stranded breaks

**DOI:** 10.1093/narmme/ugae007

**Published:** 2024-05-13

**Authors:** Huan-Yun Chen, Chia-Yu Liao, Hsun Li, Yi-Ci Ke, Chin-Hsien Lin, Shu-Chun Teng

**Affiliations:** Department of Neurology, National Taiwan University Hospital, Taipei, Taiwan; Department of Microbiology, College of Medicine, National Taiwan University, Taipei, Taiwan; Department of Microbiology, College of Medicine, National Taiwan University, Taipei, Taiwan; Department of Neurology, National Taiwan University Hospital, Taipei, Taiwan; Department of Neurology, National Taiwan University Hospital, Taipei, Taiwan; Department of Microbiology, College of Medicine, National Taiwan University, Taipei, Taiwan; Department of Neurology, National Taiwan University Hospital, Taipei, Taiwan; Institute of Molecular Medicine, College of Medicine, National Taiwan University, Taipei, Taiwan; Department of Microbiology, College of Medicine, National Taiwan University, Taipei, Taiwan; Center of Precision Medicine, National Taiwan University, Taipei, Taiwan

## Abstract

Parkinson's disease (PD) is a prevalent neurodegenerative disorder marked by the pathological accumulation of α-synuclein aggregates in dopaminergic neurons. This α-synuclein dyshomeostasis is caused by an interplay between aging, genetic and environmental factors. Aging process-related DNA damage and impaired DNA repair have recently been observed in the PD process. However, the precise neuronal response to DNA damage in PD remains largely unknown. Here, we demonstrate that double-strand breaks (DSBs) induce α-synuclein aggregation. Analysis of a large-scale proteomic analysis of ATM and ATR substrates identified a potential candidate in the HSP70 folding system responding to DNA damage. ATM phosphorylates co-chaperone DNAJB11 at threonine 188 which specifically facilitates the delivery of misfolded α-synuclein, but not tau or transthyretin protein, to the HSP70 folding system upon DSBs. Alteration of this response impairs the neurite outgrowth. Remarkably, DNAJB11 threonine 188 phosphorylation correlates with disease severity in transgenic *SNCA* mutant PD mice and PD patients. These findings reveal a DNA damage-responded HSP70 folding mechanism through a J-domain co-chaperone, offering a potential therapeutic target for PD.

## Graphical Abstract

**Graphical Abstract:**
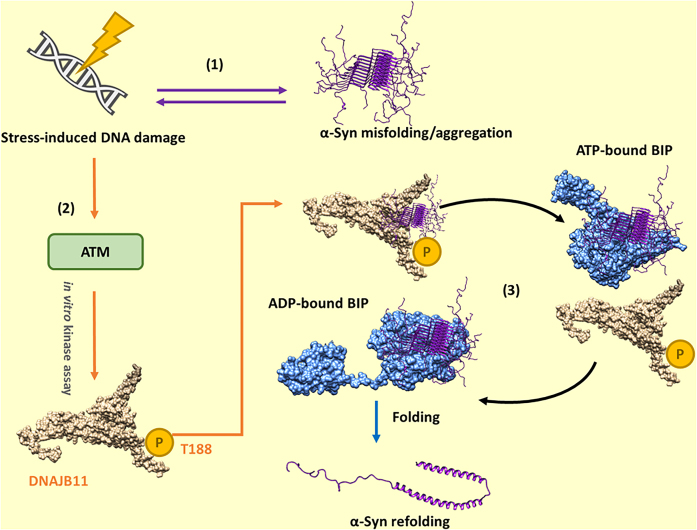

## Introduction

PD is a prevalent neurodegenerative disorder characterized by various progressive motor and non-motor symptoms. The pathognomonic features of PD are progressive loss of dopaminergic neurons in the substantia nigra of the brain resulting in dopamine insufficiency and subsequent motor dysfunction, including tremors, rigidity and bradykinesia (1). The pathological aggregation of misfolded α-synuclein protein in the dopaminergic neurons leads to the formation of Lewy bodies (LB) and promotes neurodegeneration. The exact cause of PD is not fully understood, but it is likely to involve a combination of genetic and environmental factors that cause α-synuclein misfolding and aggregation (2).

Protein folding is an intricately orchestrated and dynamic process. Chaperones, specialized proteins dedicated to aiding the proper folding of other proteins, play a crucial role in preventing misfolding and aggregation. Among these chaperones, HSP70 is the major player in the intricate duty of protein folding. Despite its folding role, HSP70 lacks client specificity (3). Contrastingly, client specificity is conferred by co-chaperones. Within the folding cycle of human HSP70, >50 diverse J-domain proteins (JDPs) deliver distinct clients to the HSP70 system (4–6). While the exact cause of PD remains largely unknown, emerging evidence points to a significant correlation between PD and DNA damage (7–10), which is a hallmark of aging. Unrepaired or incorrectly repaired DNA damage may result in severe mutations and chromosomal instability, potentially jeopardizing cell survival. A decline in DNA repair capacity and the accumulation of DNA damage can set off a cascade of events, ultimately contributing to neurodegeneration (11). Support for the causal contribution of DNA damage in the onset and progression of neurodegenerative diseases is derived from rare patients and model organisms with mutations in DNA damage-responsive genes (12). In PD, the expression of 8-OHdG, indicative of oxidative DNA damage, is notably elevated in dopaminergic neurons compared to healthy controls (13). Beyond oxidative harm, various investigations have reported a substantial increase DNA DSBs, in PD mouse models (9). Importantly, the loss of function in specific DNA repair proteins in mice can recapture the PD phenotype, and certain proteins known to play pivotal roles in PD have been demonstrated to directly mediate DNA repair (14–18). Moreover, mice lacking the DNA repair sensor ATM displayed PD-like motor abnormalities, coupled with decreased dopaminergic neuronal integrity and the presence of α-synuclein-positive inclusions (15,19). This collective evidence suggests that the alteration of several key proteins involved in DNA repair may contribute to PD.

Cells make immediate adjustments in response to various internal and external stresses. Nerve cells are constantly under the pressure of chromosomal breakage during their normal proliferation. Neuronal activity causes the formation of DNA DSBs which facilitate the expression of early-response genes (20). Furthermore, PD cells were observed to have a higher level of DNA DSBs compared to normal cells (8). However, how nerve cells cope with these pressures of DNA breakage and produce mature proteins and whether these adjustments modulate the folding of α-synuclein are not clear. Etoposide, a common chemotherapeutic drug that causes DSBs by blocking DNA topoisomerase II, triggers apoptosis at high concentrations or with extended exposure (21,22). After etoposide treatment and the onset of DNA DSBs, the phosphorylation of the serine 139 residue on the histone variant H2AX produces γH2AX through ATM (ataxia telangiectasia mutated), ATR (ATM and Rad3-related), and/or DNA-dependent protein kinase. γH2AX acts as a checkpoint for the homologous recombination and nonhomologous end-joining repair pathways (23–26). Moreover, γH2AX is a response to DNA replication fork stalling (27). Thus, etoposide serves as a valuable tool for studying DNA DSB repair signaling.

The most important cellular protein folding system is the HSP70 system. The HSP70 executes its folding cycle with the help of the JDPs, which recruit specific clients for HSP70 (4–6). In this study, we observed that DSBs induce α-synuclein aggregations. We scrutinized previously reported large-scale proteomic profiles of ATM and ATR substrates for HSP70 co-chaperones. Through functional analysis, we identified that phosphorylation of an HSP70 co-chaperone, DNAJB11, induced specifically by DSBs, reduces neuronal α-synuclein aggregation. Upon DNA DSBs, ATM kinase directly phosphorylates threonine 188 residue (T188) of DNAJB11. This phosphorylation is crucial in the delivery of α-synuclein to the HSP70 folding cycle. Notably, the DNAJB11 T188A mutation promoted α-synuclein accumulations resulting in impairing neurite outgrowth through a dominant negative effect. Moreover, the phosphorylation is boosted in a PD mouse model, as well as in post-mortem brain tissues from PD patients. These findings provide additional insights into the mechanisms of how cells adapt to DNA damage by triggering J-domain co-chaperone phosphorylation, contributing to the HSP70-mediated protein folding in PD cells.

## Materials and methods

### Cell culture and reagents

293T cells were maintained in a Dulbecco's modified Eagle's medium (DMEM) containing 10% fetal bovine serum (FBS) and antibiotics (100 U/ml penicillin and 100 μg/ml streptomycin) at 37°C with 5% CO_2_. Human neuroblastoma SH-SY5Y cells were cultured in DMEM/F12 (44.5/44.5%) medium supplemented with 10% FBS and antibiotics (100 U/ml penicillin, and 100 μg/ml streptomycin) at 37°C with 5% CO_2_. SH-SY5Y human neuroblastoma cells are one of the most widely used cellular models to study neurodegenerative diseases (28). These cells exhibit continuous proliferation, and in their undifferentiated state, they display a morphology reminiscent of neuroblasts and express immature neuronal markers. By subjecting SH-SY5Y cells to RA and neurotrophins, they can be induced to differentiate into neurons, adopting a morphology akin to primary neurons while forfeiting their proliferative capacity and expressing neuron-specific markers (29). The differentiation of SH-SY5Y cells provides a valuable tool for investigating disrupted dopamine metabolism and other pathologies specific to certain diseases (30,31). Cells were treated for the indicated time points with the final concentration of 10 μM DNA-damaging drug etoposide (Sigma, USA) (32). The ATM inhibitor, KU55933 (Selleckchem, USA), and the ATR inhibitor, AZD6738 (Selleckchem, USA), were employed at concentrations of 10 and 5 μM, respectively, 2-h before etoposide treatment. Cells were treated for 3-h with ER stress inducer tunicamycin (Sigma, USA) at the final concentration of 10 mg/ml.

### Western blotting and antibodies

Cells were harvested in cell lysis buffer and proteins were separated by 10% polyacrylamide SDS-PAGE. The target proteins were detected using the enhanced chemiluminescent reagent (GE healthcare). The antibodies used for immunoblotting were Myc (Roche, Switzerland), GFP (Santa Cruz, USA), FLAG (Sigma, USA), Phospho-CHK2 (Thr68) (Cell Signaling, USA), CHK2 (Cell Signaling, USA), His (Cytiva, USA), pSQ/TQ (Cell Signaling, USA), DNAJB11 (Proteintech, USA), Phospho-CHK1 (Ser317) (Cell Signaling, USA), CHK1 (Santa Cruz, USA), ATM (Cell Signaling, USA), HSPA8 (Novus Biologicals, USA), BIP (Abcam, UK), GST (Genetex, USA), p-AKT (S473) (Cell Signaling, USA), AKT (Cell Signaling, USA), γH2AX (upstate USA) and β-actin (Sigma, USA).

### Plasmids and cell transfection

The human DNAJB11 coding sequence was PCR-amplified, followed by cloning into the BamHI and XhoI sites of the pcDNA3.1-Myc vector and the BamHI and EcoRI sites of the pCMV-Tag2B vector, respectively. The pcDNA3.1-α-Syn^A53T^-Myc was previously described (32). The open reading frame of human tau was amplified from the Myc-tau^p301L^ plasmid and cloned into the XhoI and KpnI sites of the pEGFP-C1 vector (33). The TTR^A25T^ was a gift from Dr. R Luke Wiseman (34). pcDNA5/FRT/TO HIS DNAJB9 was acquired from Addgene. pCMV-Tag2B DNAJC10 was generated using an In-Fusion HD Cloning Kits (Takara, Japan). The WT or mutant DNAJB11 was PCR-amplified and cloned into the pET-28a vector at the BamHI and HindIII sites. The full-length human α-synuclein was cloned into the pGEX-6P-2 vector at the XmaI and NotI sites. The WT or mutant DNAJA3 was PCR-amplified and cloned into the pcDNA3.1-Myc vector. The pcDNA3.1/Myc-His-ErbB2 plasmid was kindly provided by Dr. Ming-Shyue Lee (35). 293T cells were transfected with T-Pro Non-liposome Transfection Reagent (T-Pro Biotechnology) and SH-SY5T cells were transfected with Lipofectamine LTX Reagent (Thermo Fisher Scientific, USA) according to the manufacturer's instructions.

### Filter-trap assay

293T and SH-SY5Y cells were treated with 10 μM of etoposide and transfected with DNAJB11 WT or mutant plasmids. Cell pellets were collected and lysed with a buffer containing 50 mM Tris–HCl pH 7.5, 150 mM NaCl, 2 mM EDTA, 0.1% NP40 and 1 mM PMSF supplemented with protease inhibitors (Roche, Switzerland) and PhosSTOP Phosphatase Inhibitors (Roche, Switzerland). The samples were mixed with SDS to a final concentration of 2% and filtrated through a 96-well dot blot apparatus (Bio-Rad Laboratories, USA) containing a 0.2 μm nitrocellulose membrane. The nitrocellulose membrane was then probed with the anti-α-synuclein antibody (Genetex, USA), anti-Myc antibodies (Roche, Switzerland), anti-GFP (Santa Cruz, USA) antibodies, and anti-FLAG antibodies (Sigma-Aldrich, USA). Chemiluminescence was quantified using the ImageJ software.

### Co-IP

Etoposide-treated cells were harvested in NP40 lysis buffer (50 mM Tris–HCl pH 7.5, 150 mM NaCl, 2 mM EDTA, 0.1% NP40, and 1 mM PMSF) supplemented with protease inhibitors (Roche, Switzerland) and PhosSTOP Phosphatase Inhibitors (Roche, Switzerland). Cell lysates (1000 μg) and human brain lysates (200 μg, Novus Biological) were incubated overnight with anti-DNAJB11 (2 μg, Proteintech, USA), anti-FLAG antibodies (3.8–4.2 μg, Sigma-Aldrich, USA) or anti-Myc antibodies (1 μg, Roche, Switzerland) at 4°C. Immunocomplexes were isolated with protein A-Sepharose beads saturated with 1% BSA, by rotating for 5-h at 4°C. After incubating and washing the mixture, bound proteins were denatured, eluted, and resolved by 10% polyacrylamide SDS-PAGE.

### Purification of the His6-DNAJB11 recombinant proteins

The expression of the His-DNAJB11, His-DNAJB11-T188A, or T188E was performed in *Escherichia coli* BL21 (DE3) tRNA cells by induction with 0.1 mM IPTG overnight at 16°C. The cells were harvested by centrifugation and suspended in 20 mM Tris–HCl buffer (pH 7.4), containing 0.22 mM imidazole, 200 mM NaCl, 1 mg lysozyme and 100 mM PMSF. The cells were lysed by sonication and cleared by centrifugation. The clarified lysates were isolated with Talon beads by rotating for 2-h at 4°C. After incubating and washing the mixture, His6-tagged DNAJB11 WT or mutant proteins were eluted with 20 mM Tris–HCl buffer (pH 7.4), containing 0.5 mM imidazole, 0.22 mM NaCl and 0.5% SDS.

### *In vitro* kinase assay

For the *in vitro* kinase assay, 293T or SH-SY5Y cells treated with etoposide and/or ATM inhibitor were lysed in TENG buffer (150 mM NaCl, 100 mM Tris pH 7.4, 10% glycerol and 0.05% NP40) containing protease inhibitors (Roche, Switzerland) and 1 mM DTT. The lysates were sonicated followed by centrifugation at 16 200 g for 20 min. IP of ATM was performed by incubating the lysates with protein A-Sepharose beads for 5 h at 4°C. The beads were washed three times with TEGN buffer, two times with TEGN buffer supplemented with 700 mM LiCl, and finally, two times with kinase buffer (20 mM Tris pH 7.4, 10 mM MgCl_2_, 2 mM MnCl_2_, 1 mM PMSF, 1 mM DTT and 10 mM NaF) supplemented with protease inhibitors (Roche, Switzerland). Purified recombinant WT or mutant DNAJB11 were incubated with ATM binding beads in kinase buffer in the presence of 50 μM ATP and for 15 min at 30 °C. The kinase reaction was stopped in 5× SDS loading dye (5% β-mercaptoethanol, 0.02% bromophenol blue, 30% glycerol, 10% SDS, and 250 mM Tris pH 6.8). The reaction was analyzed by western blotting.

### Immunofluorescence and confocal microscopy

Stress-induced cells or cells transfected with DNAJB11 WT, T188A or T188E, with or without A53T α-synuclein, were seeded onto glass coverslips (Marienfeld Laboratory Glassware, Germany) at 4 × 10^5^ cells/ml in 6-well plates and fixed with 4% paraformaldehyde in PBS for 20 min at room temperature. Fixed cells were washed with PBS and permeabilized with 0.1% Triton X-100 in PBS for 5 min. After washing with PBS, the coverslips were incubated with anti-Myc (Roche, Switzerland), anti-ATM (Cell Signalling, USA), anti-BIP (Abcam, USA), and anti-α-synuclein (Genetex, USA) specific antibodies overnight at 4°C. The coverslips were incubated with Fluorescein (FITC) AffiniPure Goat Anti-Mouse IgG (H + L) (Jackson ImmunoResearch, USA), Rhodamine Red-X-conjugated goat-anti-rabbit IgG (H + L) (Jackson ImmunoResearch, USA) and Donkey anti-Rat IgG (H + L) Highly Cross-Adsorbed Secondary Antibody, Alexa Fluor™ 647 (Thermo Fisher Scientific, USA) for overnight at 4°C. After washing twice with PBS, the coverslips were stained with DAPI for 10 minutes, and cells were mounted with a mounting medium (Sigma, USA). Confocal images were captured under the Zeiss LSM880 confocal microscope. The percentage of cells with α-synuclein foci was determined by counting at least 40 cells in five randomly chosen fields using the ImageJ software. In each cell, a mask for the region of interest (ROI) excluding the nucleus was generated by ImageJ, and pixel intensities of DNAJB11 or ATM immunofluorescence within the ROI were recorded. Thresholds for each immunofluorescence were set as threefold standard variations higher than mean intensities respectively. Subsequently, pixels with intensity values higher than thresholds were taken into consideration, and the percentages of DNAJB11 and ATM colocalized were quantified.

### Duolink PLA

PLA was performed using the Duolink In Situ Red Starter Kit Mouse/Rabbit (Sigma-Aldrich, USA), and all reagents used for the procedure are from this kit. SH-SY5Y cells were transfected with DNAJB11 WT, T188A or T188E along with A53T α-synuclein, and seeded onto glass coverslips (Marienfeld Laboratory Glassware, Germany) at 1 × 10^4^ cells/ml in 12-well plates and fixed with 4% paraformaldehyde in PBS for 15 min at room temperature. Fixed cells were washed with PBS and permeabilized with 0.1% Triton X-100 in PBS for 5 min. Coverslips were blocked with 1x Duolink blocking solution for 30 min. Anti-FLAG and anti-Myc antibodies were then added (20 μg/ml in Duolink antibody diluent) and slides were incubated at 4°C overnight. Following incubation, the slides were incubated with PLA PLUS and PLA MINUS probes (diluted 1:5) for 37°C 1-h. The PLA probes are secondary anti-mouse or anti-rabbit antibodies conjugated to unique DNA oligonucleotides (the sequence of which is not shared by the manufacturer). After another washing step in the washing buffer, ligase was added (1:40 dilution in ligase solution), and the slides were incubated for 30 minutes to allow for the ligation and circularization of the DNA-oligos. Finally, the coverslips were washed with wash buffer A, and incubated with DNA polymerase (1:80 dilution in amplification solution) for 100 minutes. Following incubation, the slides were washed again with wash buffer B, and the slides were then mounted with one drop of Duolink *in situ* mounting medium containing DAPI to stain the nuclei, and analyzed with a confocal microscope (Zeiss LSM880).

### GST pull-down assay

pGEX-6P-2-α-synuclein (GST-tagged), pET-28a-DNAJB11 WT, T188A or T188E (His6-tagged) plasmids were transformed into *E. coli* BL21 (DE3) strains, respectively, and cultured overnight. The bacteria were 1:100 subcultured into LB (100 μl to 10 ml) containing 100 μg/ml ampicillin or kanamycin and then incubated at 37°C with vigorous agitation (200 rpm) until OD_600_ reached 0.6–0.8. The expression of the recombinant proteins was induced by 0.1 mM IPTG at 16°C overnight. The bacterial broth was centrifuged at 10 000 g for 5 min. The pellets were washed in ice-cold PBS (137 mM NaCl, 2.7 mM KCl, 4.3 mM Na_2_HPO_4_ and 1.45 mM KH_2_PO_4_) and lysed in lysis buffer (1% Triton-X100, 1 mM EDTA, 1 mM DTT, 1 mM PMSF and 1 mg/ml lysozyme in PBS) containing protease inhibitor (Roche, Switzerland). Lysates with GST-α-synuclein and His6-DNAJB11 were co-incubated with glutathione Sepharose™ beads (GE healthcare, USA) at 4°C in binding buffer (0.5% NP40 in PBS) on an end-to-end rotator (10 rpm) for 1-h. The beads were washed three times with 500 μl binding buffer to remove unbound proteins. The precipitated proteins were eluted by boiling with 50 μl of 2 SDS loading dye (5% β-mercaptoethanol, 0.02% bromophenol blue, 30% glycerol, 10% SDS, and 250 mM Tris pH 6.8) and separated by SDS-PAGE.

### Analysis of neurite outgrowth

1 × 10^4^ cells were grown in 6-well plates coated with 50 μg/ml poly-d-lysine (A3890401, Gibco). Cells were differentiated into a neuronal phenotype by exposing them to 10 μM all-trans RA (Sigma Aldrich, USA) in a culture medium with 2% of FBS for 7 days (36). Images of differentiated SH-SY5Y cells (four images per well of a 6-well plate) were acquired after differentiation using ZEISS Axio Observer 7. Neurite lengths were manually quantified in differentiated SH-SY5Y cells using Image J software as described previously (36).

### Co-IP of transgenic *SNCA* p.A53T mice

The transgenic *SNCA* p.A53T mice (B6.Cg-2310039L15RikTg(Prnp-SNCA*A53T)23Mkle/J) (37) were examined for the phosphorylation of DNAJB11. These mice express a missense mutant form of human α-synuclein (*SNCA* gene), specifically the A53T variant, regulated by the murine prion promoter, resulting in the expression of the aggregation-prone mutant α-synuclein protein (37,38). Hemizygous mice develop adult-onset age-dependent locomotor dysfunction between 6 and 8 months of age. This motor dysfunction is evident in tasks such as beam walking, rotarod, and open field tests. Offspring were tail-genotyped and maintained in the specific pathogen-free facility. Brains of transgenic *SNCA* p.A53T mice and littermate control mice were harvested in NP40 lysis buffer (50 mM Tris–HCl pH 7.5, 150 mM NaCl, 2 mM EDTA, 0.1% NP40 and 1 mM PMSF) supplemented with protease inhibitors (Roche, Switzerland) and PhosSTOP phosphatase Inhibitors (Roche, Switzerland). Mouse brain lysates (1000 μg) were incubated overnight with anti-DNAJB11 (2 μg, Proteintech, USA) at 4°C. Immunocomplexes were isolated with protein A-Sepharose beads saturated with 1% BSA by rotating for 3-h at 4°C. After incubating and washing the mixture, bound proteins were denatured and resolved by 10% polyacrylamide SDS-PAGE.

All animal studies were performed according to the guidelines and approved by the Institutional Animal Care and Use Committee (IACUC) of the College of Medicine of National Taiwan University Hospital with the approval number 20230080.

### Collection and analysis of GEO profile data

The dataset from GDS2821, comprising 9 specimens from normal substantia nigra and 16 specimens from substantia nigra affected by Parkinson's disease, was downloaded from GEO (https://www.ncbi.nlm.nih.gov/geo/tools/profileGraph.cgi?ID = GDS2821:212672_at). This dataset was generated using Affymetrix Human Genome U133 Plus 2.0 GeneChip arrays and was submitted by Lesnick, Timothy G. *et al.* in 2007. The data in this profile are associated with the Kyoto Encyclopedia of Genes and Genomes (KEGG) (39).

The microarray dataset for α-synuclein deficiency mice, GDS4153, was obtained from the GEO database (https://www.ncbi.nlm.nih.gov/geo/tools/profileGraph.cgi?ID = GDS4153:1423151_at). In this dataset, cerebellum samples from wild-type (WT) mice (*n* = 4) and *SNCA* knockout mice (*n* = 4) at both 6 and 21 months of age, as well as striatum samples from WT mice (*n* = 3) and *SNCA* knockout mice (*n* = 3) at the same ages, were utilized. The data were analyzed using Expression Console 1.0 (Affymetrix) and the Bioconductor package ‘affy’ in R version 2.7.0. Background correction, normalization, and probe summarization were performed using the Robust Multi-array Average (RMA) method (https://www.rdocumentation.org/packages/affy/versions/1.50.0/topics/justRMA) (40,41).

### Prediction of molecular structures

The DNAJB11 WT protein structure is obtained from the AlphaFold Protein Structure Database (https://alphafold.ebi.ac.uk/entry/Q9UBS4). The three-dimensional structures of the proteins are represented here using the UCSF Chimera package from the Resource for Biocomputing, Visualization, and Informatics at the University of California, San Francisco (http://www.cgl.ucsf.edu/chimera/) (accessed on 24 September 2023).

### Statistical analysis

All experiments were performed with at least three biological repeats. Paired data were expressed as means ± standard deviation (SD) and analyzed using the Student's *t*-test with the two-tailed distribution.

## Results

### DSB-induced DNAJB11 T188 phosphorylation reduces neuronal α-synuclein aggregation

Nerve cells experience continuous chromosomal breakage during their normal proliferation, a process intensified by neuronal activity and during the aging process, leading to the creation of DNA DSBs, which plays a role in facilitating the expression of early-response genes (20). In the context of PD, nerve cells exhibit even higher levels of DNA DSBs (8). Nevertheless, the mechanisms by which nerve cells manage these DNA breakage challenges to produce properly folded proteins remain unclear. Additionally, it is uncertain whether these environmental changes influence the folding of α-synuclein. To test this, we first treated cells with a DSB-inducing agent, etoposide. Notably, etoposide treatment induced α-synuclein aggregation in both human embryonic kidney 293T and neuroblastoma SH-SY5Y cells (Supplementary Figure S1A and S1B), suggesting a crosstalk between DNA damage and α-synuclein proteostasis.

To identify α-synuclein-specific protein folding pathways in response to DNA damage, we searched for HSP70 co-chaperones in a previously reported large-scale proteomic analysis of ATM and ATR substrates (42) (Supplementary Figure S1C). In the database, two HSP70 co-chaperones exhibit potential ATM/ATR phosphorylation sites. We subsequently conducted different assays for each candidate to determine the function of these phosphorylation sites, including DNAJA3 serine (S) 169 and DNAJB11 T188, on proteostasis. ErbB2, a member of the ErbB receptor tyrosine kinase family, is observed in up to 30% of primary breast cancers (43). Previous reports have shown that increased expression of DNAJA3 suppresses the expression level of ErbB2 via the ubiquitin-proteasome pathway (44). To dissect the importance of DNAJA3 S169 phosphorylation in controlling proteostasis, we assessed the ErbB2 expression levels in the presence of etoposide in DNAJA3 wild-type (WT), dephospho-mimicking alanine (A), and phospho-mimicking aspartic acid (D) mutant expressing 293T cells. Ectopic expression of DNAJA3 WT increased ErbB2 degradation, but DNAJA3 S169A-expressing did not impair the DNAJA3-promoted ErbB2 degradation (Supplementary Figure S1D and S1E). We further evaluated the involvement of DNAJB11 T188 phosphorylation in the folding of misfolded proteins under etoposide treatment.

**Figure 1.**
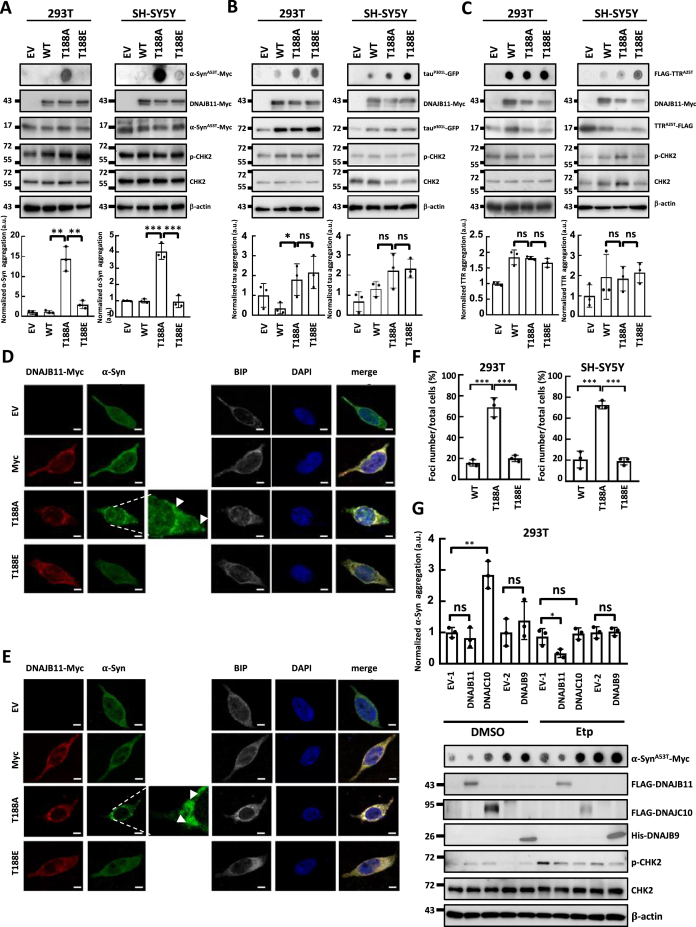
DNAJB11 T188A mutation induces α-synuclein aggregation. (A–C) DNAJB11 T188A mutant increases SDS-insoluble aggregation of α-synuclein in 293T and SH-SY5Y cells. 293T and SH-SY5Y cells were transfected for 72-h with WT, mutant DNAJB11, andα-synuclein^A53T^ mutant (α-Syn^A53T^) (**A**), tau^P301L^ (**B**) or TTR^A25T^ (**C**). 24-h after transfection, cells were treated with 10 μM of etoposide (Etp) for 48-h or solvent DMSO. Aggregates were detected by the filter-trap assay in cells transfected with the DNAJB11 WT, T188A, or T188E plasmid. The lysate was diluted in SDS and filtered through nitrocellulose membranes. α-Syn^A53T^-Myc (A), tau^P301L^-GFP (B) or FLAG-TTR^A25T^ (C) immunostaining was detected by the specific antibodies, respectively. A representative image and the densitometry data are shown (a.u., arbitrary unit). The values of Syn^A53T^-Myc, tau^P301L^-GFP or FLAG-TTR^A25T^ aggregates were normalized to the number of aggregates in the empty vector control (top panel). Ectopic expression of DNAJB11 WT, T188A, or T188E was examined by Western blot analysis (bottom panel). (**D, E**) Images of 293T (**D**) and SH-SY5Y (**E**) cells following DNAJB11 WT, T188A, or T188E ectopic expression and etoposide treatment were captured. Scale bar, 20 μm. (**F**) Quantified results in (D, E) are shown as the percentage of cells with α-synuclein aggregated foci. (**G**) DNAJB9 and DNAJC10 do not decrease α-synuclein aggregation in SH-SY5Y cells. α-Syn^A53T^-Myc aggregation was detected by the filter-trap assay in cells transfected with the DNAJB9, DNAJC10 or DNAJB11 plasmid. The lysate was diluted in SDS and filtered through nitrocellulose membranes. α-synuclein immunostaining was detected by the anti-Myc antibody. A representative image and the densitometry data are shown. The values of α-Syn^A53T^-Myc aggregation were normalized to the amount of aggregation in the empty vector control. Error bars represent the SD of the means calculated using data from three independent experiments (Student's *t*-test, *P < 0.05, **P < 0.01, ***P < 0.001).

The aggregation of α-synuclein is implicated in PD pathogenesis. It is widely believed that its abnormal soluble oligomeric conformations, referred to as protofibrils, are the toxic entities responsible for disrupting cellular homeostasis and triggering neuronal death (45,46). Tau, a microtubule-associated protein, forms insoluble filaments that accumulate as neurofibrillary tangles in AD and related tauopathies (47,48). The aggregation of transthyretin (TTR) results in the formation of amyloid fibrils and it has been reported that monomer unfolding is the underlying cause of life-threatening transthyretin amyloidosis (49,50). We, therefore, conducted a filter trap assay to screen for the levels of SDS-insoluble α-synuclein, tau and TTR aggregates. The T188E mutation, which involves changing T to phosphor-mimicking glutamic acid (E), was employed to explore the functional significance of the ATM/ATR-targeting S/TQ site (42) by simulating the potential impact of phosphorylation on the protein's activity. Remarkably, compared to DNAJB11 WT-expressing cells, α-synuclein aggregation increased in T188A-expressing cells but recovered in T188E-expressing 293T and SH-SY5Y cells (Figure 1A). On the other hand, T118A-induced aggregation along with T188E-displayed recovery were not observed in tau and TTR aggregations (Figure 1B and C). ATM phosphorylates CHK2 at threonine 68 upon etoposide treatment (51). Therefore, we detected phosphorylated CHK2 at threonine 68 as an indicator of ATM activation. Collectively, these data suggest that the HSP70-related co-chaperone DNAJB11 may function as a potential DNA DSB-associated co-chaperone that specifically mediates the folding of α-synuclein.

To further investigate the potential effects of the DNAJB11 T188A mutation on α-synuclein aggregation, we visualized α-synuclein protein aggregation using confocal microscopy. The α-synuclein aggregated foci increased in DNAJB11 T188A-expressing cells (Figure 1D and E). Consistently, a substantial reduction of α-synuclein aggregates was observed in T188E-expressing cells compared to WT (Figure 1D, F). These findings suggest that the DNAJB11 T188A mutation leads to α-synuclein aggregation.

DNAJB11 was initially recognized as a JDP that aids in alleviating endoplasmic reticulum (ER) stress, which results from disruptions in protein folding within the ER (34,52–54). Aside from DNAJB11, DNAJC10 and DNAJB9 are the other two JDPs induced under ER stress and play crucial roles in unfolded protein response (UPR) to mitigate ER stress by promoting proper protein folding and preventing protein aggregation (55). Hence, in addition to DNAJB11, we also employed the filter-trap assay to determine the ability of the other ER stress-induced JDPs to resolve the SDS-insoluble α-synuclein aggregation. Expression of neither DNAJB9 nor DNAJC10 reduced the levels of SDS-insoluble α-synuclein aggregates in 293T cells following etoposide treatment (Figure 1G). Conversely, only the ectopic expression of DNAJB11 reduced SDS-insoluble α-synuclein aggregates under etoposide treatment. Together, these results suggest that DNAJB11 is a specific ER stress-induced JDP that alleviates α-synuclein aggregation under etoposide treatment.

### DNAJB11 T188Q is phosphorylated upon DNA DSBs

**Figure 2.**
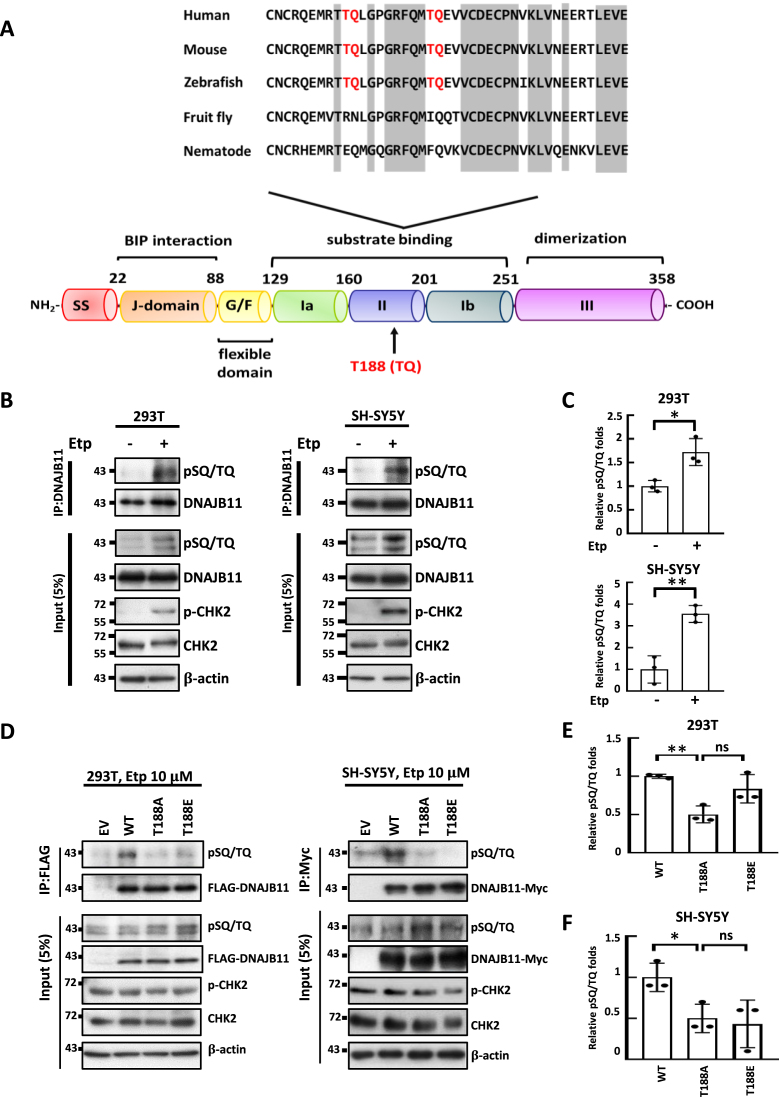
DNA DSBs induce DNAJB11 T188 phosphorylation. (**A**) Image showing the domain architecture of DNAJB11 including the cleavable N-terminal signal sequence (ss), J-domain, flexible Gly/Phe-rich domain (G/F), bifurcated substrate binding domain I (Ia and Ib), Cys-rich domain II, and C-terminal dimerization domain III. Sequence alignment (top panel) demonstrates the evolutionary conservation of the ATM/ATR substrate motif T188Q in DNAJB11 protein across the indicated species. The conservation of residues is labeled in gray, and T178Q and T188Q are marked in red. (**B**) Immunoprecipitation (IP) analysis of DNAJB11 phosphorylation. 293T and SH-SY5Y cells were treated with 10 μM of etoposide (Etp) for 48-h. IPs were performed with an anti-DNAJB11 antibody. Immunoprecipitates were sequentially probed with anti-pSQ/TQ antibodies. Five percent of lysates used for IP were loaded as input and probed with anti-pSQ/TQ, anti-DNAJB11, anti-pCHK2, and anti-CHK2 antibodies. β-actin was used as a loading control. (**C**) Quantification of the ratio of pSQ/TQ to immunoprecipitated DNAJB11 in (B). The amounts of pSQ/TQ and DNAJB11 were quantified, using the Image J software. The results of the quantitative analysis are shown as the relative values to the DMSO control. (**D**) Etoposide treatment induces DNAJB11 T188 phosphorylation. 293T and SH-SY5Y cells transiently expressing an empty vector or a vector encoding FLAG- or Myc-tagged DNAJB11, T188A, and T188E were treated with etoposide for 48-h. IPs were performed with an anti-FLAG or anti-Myc antibody. (**E**, **F**) Quantification of the ratio of pSQ/TQ to immunoprecipitated FLAG-tagged or Myc-tagged DNAJB11 in (D). Immunoprecipitates were sequentially probed with anti-pSQ/TQ and anti-FLAG or anti-Myc. Five percent of lysates used for IP were loaded as the inputs and probed with anti-pSQ/TQ, anti-pCHK2, anti-CHK2, anti-FLAG, or anti-Myc antibodies. β-actin is a loading control (Student's *t*-test, *P < 0.05, **P < 0.01).

DNAJB11 is a JDP with a well-defined domain architecture, featuring a cleavable N-terminal signal sequence (ss), a J-domain, a flexible Gly/Phe-rich domain (G/F), bifurcated substrate binding domain I (Ia and Ib), a Cys-rich domain II, and a C-terminal dimerization domain III (Figure 2A, bottom panel) (52). The T188-Q189 motif is conserved from zebrafish to human DNAJB11 homologs and is situated within the substrate binding domain, directly responsible for binding unfolded proteins (Figure 2A and Supplementary Figure S2). The presence of the T188Q site within the conserved domain of DNAJB11 implies that this phosphorylation may alter the function of DNAJB11 (56). Given that the DNAJB11 sequence contains two S/TQ motifs (T178Q and T188Q) (Supplementary Figure S2) and the previous mass spectrometry studies only detected the phosphorylation of DNAJB11 at T188Q upon irradiation (42), we specifically focused on T188Q, which is highly conserved in vertebrates (Figure 2A and Supplementary Figure S2).

To detect DNAJB11 phosphorylation in response to DNA damage, we exposed epithelial-like 293T and neuroblastoma SH-SY5Y cells to DNA-damaging agent etoposide (Figure 2B) and ER stress inducer tunicamycin (Supplementary Figure S3). Western blot analysis using antibodies against ATM/ATR substrates (pS/TQ) (57) revealed a substantial increase in DNAJB11 phosphorylation following etoposide treatment but not in tunicamycin-treated cells (Figure 2B, C and Supplementary Figure S3). The lower band in the immunoblot data (Supplementary Figure S3) corresponds to the aglycosylated form of DNAJB11 induced by ER stress (58). To further validate that T188Q is the major phosphorylation site in DNAJB11 responsive to etoposide treatment, we expressed WT and two DNAJB11 mutants T188A and T188E in 293T and SH-SY5Y cells. Western blot analysis of the immunoprecipitated DNAJB11 demonstrated that etoposide-induced DNAJB11 TQ phosphorylation was greatly reduced in DNAJB11-T188A or T188E mutants (Figure 2D–F), indicating that T188Q is the primary TQ phosphorylation site of DNAJB11 in response to etoposide treatment.

### ATM directly phosphorylates DNAJB11 at T188 in response to DSBs

**Figure 3.**
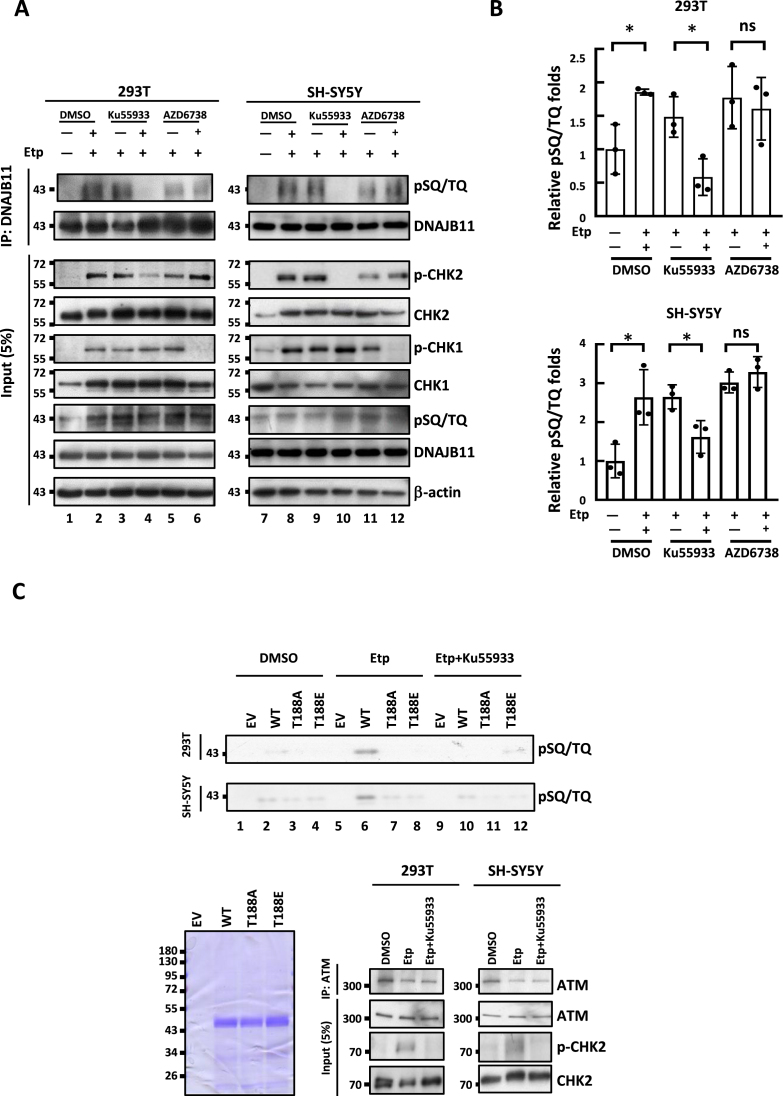
Etoposide induces ATM-dependent DNAJB11 T188 phosphorylation. (**A**) Etoposide-induced DNAJB11 phosphorylation is reduced in ATM inhibitor-treated cells. 293T and SH-SY5Y cells were pretreated with the ATM-specific inhibitor Ku55933, ATR-specific inhibitor AZD6738, or solvent DMSO for 24-h, followed by treatment with 10 μM of etoposide for 24-h. IPs were performed with an anti-DNAJB11 antibody. Immunoprecipitates were sequentially probed with anti-pSQ/TQ and anti-DNAJB11 antibodies. Five percent of lysates used for IP were loaded as the inputs and probed with anti-pSQ/TQ, anti-pCHK2, anti-CHK2, anti-pCHK1, anti-CHK1 and anti-DNAJB11 antibodies. β-actin is a loading control. (**B**) The amounts of pSQ/TQ and DNAJB11 were quantified, using the ImageJ software. The results of the quantitative analysis are shown as the relative values to the solvent controls (Student's *t*-test, *, *P* < 0.05). (**C**) ATM phosphorylates DNAJB11 T188 *in vitro*. 293T and SH-SY5Y cells were pretreated with or without the ATM-specific inhibitor Ku55933 and followed by treatment with or without 10 μM of etoposide. *In vitro* ATM kinase assay was conducted using immunoprecipitants of ATM on recombinant His6-DNAJB11, His6-DNAJB11 T188A, and His6-DNAJB11 T188E substrates. Samples were loaded onto a 10% SDS-PAGE, and the phosphorylated proteins were detected by anti-pSQ/TQ antibodies (upper panel, *n* = 3). The same samples were stained with Coomassie blue to confirm that all proteins were equally loaded (left panel). The precipitated ATM kinases were resolved by a 6% SDS-PAGE and detected with anti-ATM antibodies (right panel, *n* = 3).

DNA damage triggers the activation of ATM and ATR kinases, two integral components of the cellular response in preserving genomic integrity (59). To investigate whether etoposide-induced phosphorylation of DNAJB11 is regulated by ATM or ATR, we compared the phosphorylation status of DNAJB11 in cells treated with the ATM inhibitor KU-55933 or the ATR inhibitor AZD6738 after etoposide treatment. Immunoblotting analysis revealed etoposide-induced DNAJB11 phosphorylation in both 293T and SH-SY5Y cells compared to the untreated controls (Figure 3A and B). The phosphorylation of DNAJB11 was reduced by the ATM inhibitor KU-55933 but not by the ATR inhibitor AZD6738, indicating the specificity of ATM in this regulation (Figure 3A and B).

To further examine whether DNAJB11 is a direct ATM substrate, an *in vitro* kinase assay was conducted. Immunoprecipitated ATM from etoposide-treated cells could phosphorylate *Escherichia coli* expressed and purified full-length DNAJB11 (Figure 3C, upper panel, lanes 6). In parallel, phosphorylation was essentially absent in both the T188A and T188E mutants (Figure 3C, upper panel, lanes 7 and 8). ATM-mediated DNAJB11 phosphorylation was completely repressed by ATM inhibitor KU-55933 treatment, confirming the ATM’s role in DNAJB11 T188 phosphorylation under etoposide treatment (Figure 3C, upper panel, lanes 9–12). These findings suggest that ATM directly phosphorylates DNAJB11 T188 residue following DSBs.

### DNAJB11 and ATM colocalize upon DSBs

**Figure 4.**
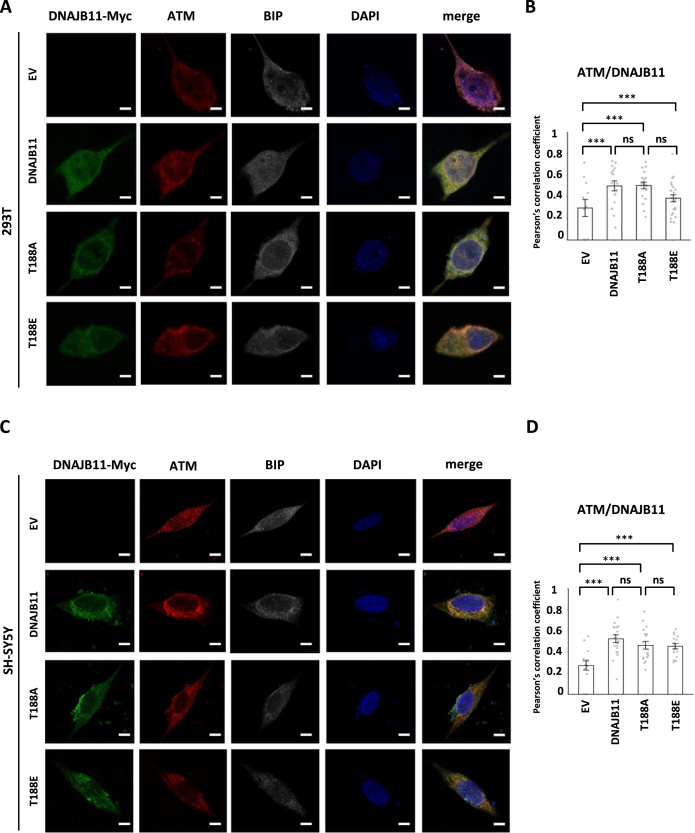
DNAJB11 and ATM co-localize in DNAJB11-Myc ectopic expression cells. (**A, C**) After 48-h of pretreatment with 10 μM of etoposide, Myc-tagged DNAJB11, T188A, or T188E expressing 293T and SH-SY5Y cells were paraformaldehyde-fixed and stained with anti-Myc (green), anti-ATM (red), anti-BIP (white) antibodies, and DAPI (blue) was used to stain the nuclear DNA. The images (1260 x) were acquired using the Zeiss LSM880 confocal microscope. The scale bar shows 20 μm. (**B**, **D**) Percentages of co-localized DNAJB11 and ATM in (A) 293T and (C) SH-SY5Y cells transfected with control pCDNA3.1, DNAJB11, T188A, or T188E expressing vectors were quantified. Bar graphs represent means ± standard deviation (SD). Comparisons between two data groups were processed by Student's *t*-tests, and the statistical significance was shown as ***P < 0.001.

ATM acts as a master regulator of cell-cycle checkpoints and DNA DSB repair pathways, coordinating responses to specific DNA damage (25). While ATM is primarily a nuclear protein, previous studies have indicated the presence of cytoplasmic ATM, which is required to prevent lysosomal accumulation and modulate synaptic function (60). Additionally, DNAJB11 and BIP reside in both cytosol and ER (61–65). To investigate the subcellular localization of ATM and DNAJB11 in response to etoposide, we observed the intracellular distribution of ATM and DNAJB11 in etoposide-induced and DNAJB11 WT- and mutant-expressing 293T and SH-SY5Y cells using immunostaining and confocal microscopy. Compared to solvent-treated control cells, ATM and DNAJB11 were enriched in the majority of stress-exposed cells. Additionally, merged images revealed colocalization of ATM and DNAJB11 in the cytosolic regions (Figure 4A and C). Quantitative analysis demonstrated that, following the overexpression of DNAJB11 WT or mutants, there were significant increases in the proportions of cells exhibiting colocalization of ATM and DNAJB11 WT or mutants (Figure 4B and D), suggesting that ATM and DNAJB11 may colocalize in cells.

### DNAJB11 T188 phosphorylation promotes the binding of α-synuclein but does not change its interaction with BIP

**Figure 5.**
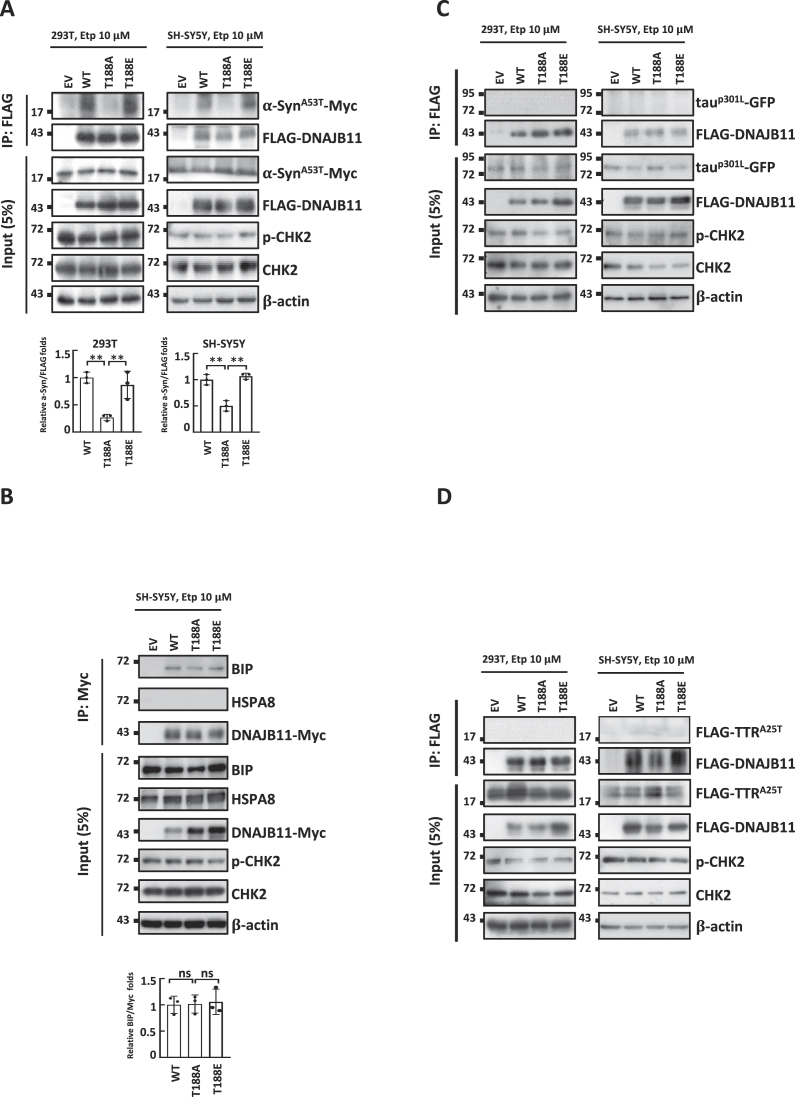
DNAJB11 T188 phosphorylation enhances the binding between DNAJB11 and misfolded α-synuclein. (**A**) 293T and SH-SY5Y cells transiently expressing FLAG-tagged DNAJB11 (WT or mutants) and Myc-taggedα-Syn^A53T^ mutant were treated with etoposide for 48-h. IPs were performed with an anti-FLAG antibody. Immunoprecipitates were sequentially probed with anti-Myc and anti-FLAG antibodies. Five percent of lysates used for IP were loaded as the inputs and probed with anti-Myc, anti-pCHK2, and anti-CHK2 antibodies. β-Actin is a loading control. The amounts of Myc and FLAG were quantified, using the Image J software. The results of the quantitative analysis are shown as the relative values to the WT. (**B**) SH-SY5Y cells transiently expressing Myc-tagged DNAJB11 (WT or mutants) were treated with etoposide for 48-h. IPs were performed with an anti-Myc antibody. Immunoprecipitates were sequentially probed with anti-Myc, anti-BIP, and anti-HSPA8 antibodies. (C, D) DNAJB11 T188 phosphorylation is not involved in misfolded tau (**C**) and TTR (**D**) clearance. IP analysis was performed with cells expressing an empty vector or a vector encoding FLAG-tagged DNAJB11, T188A and T188E along with tau^P301L^ or TTR^A25T^, followed by treatment with 10 μM of etoposide for 48-h. Immunoprecipitates were sequentially probed with anti-GFP or anti-FLAG antibodies. Five percent of lysates used for IP were loaded as the inputs (Student's *t*-test, **P< 0.01).

T188Q is located within the substrate binding domain of DNAJB11. Additionally, mutant α-synuclein (A53T) is known for its propensity to misfold and DNAJB11 was identified in the p.A53T proteome (66). These prompted us to evaluate whether etoposide-induced DNAJB11 T188 phosphorylation contributes to α-synuclein recruitment to the HSP70/BIP folding system in neuronal cells. We hypothesized that mutant α-synuclein (A53T) would interact with DNAJB11 in cells. To test this, 293T and SH-SY5Y cells were transfected with FLAG-tagged WT or mutants DNAJB11 along with mutant α-synuclein (A53T) and then treated with etoposide, followed by co-immunoprecipitation (co-IP). Interestingly, DNAJB11 WT and DNAJB11T188E mutant, but not DNAJB11 T188A mutant, co-immunoprecipitated α-synuclein^A53T^ (Figure 5A). These results indicate that DNAJB11 T188 phosphorylation enhances the DNAJB11-α-synuclein interaction.

BIP, a pivotal HSP70 chaperone protein mainly situated in the ER but also distributed in the cytoplasm, has a critical function in protein folding, assembly, and quality control, ensuring the correct performance of secretory and transmembrane proteins (67). DNAJB11 is a co-chaperone that partners with BIP by interacting through its J-domain, which binds to the ATPase domain of BIP. This interaction enables DNAJB11 to enhance the ATPase activity of BIP and facilitate protein folding (53,65). To examine whether DNAJB11 T188 phosphorylation is involved in the interaction with BIP, we conducted a co-IP assay to examine the interaction between DNAJB11 and BIP. Notably, DNAJB11 wild-type (WT) and T188 mutants exhibited interaction with BIP but not with HSPA8, the major cytoplasmic HSP70 (Figure 5B). The co-IP efficiency is similar between DNAJB11 WT and T188 mutants, suggesting that DNAJB11 T188 phosphorylation does not change the interaction between DNAJB11 and BIP (Figure 5B).

We further examined the association between DNAJB11 and tau^P301L^ or TTR^A25T^. In line with the findings from the tau^P301L^ and TTR^A25T^ filter trap assays (Figure 1B and C), there was no apparent interaction between DNAJB11 and tau^P301L^ or TTR^A25T^ (Figure 5C and D). These results indicate that tau and TTR may not be the clients of DNAJB11 co-chaperone.

### Phosphorylated DNAJB11 interacts with α-synuclein in neuronal cells

**Figure 6.**
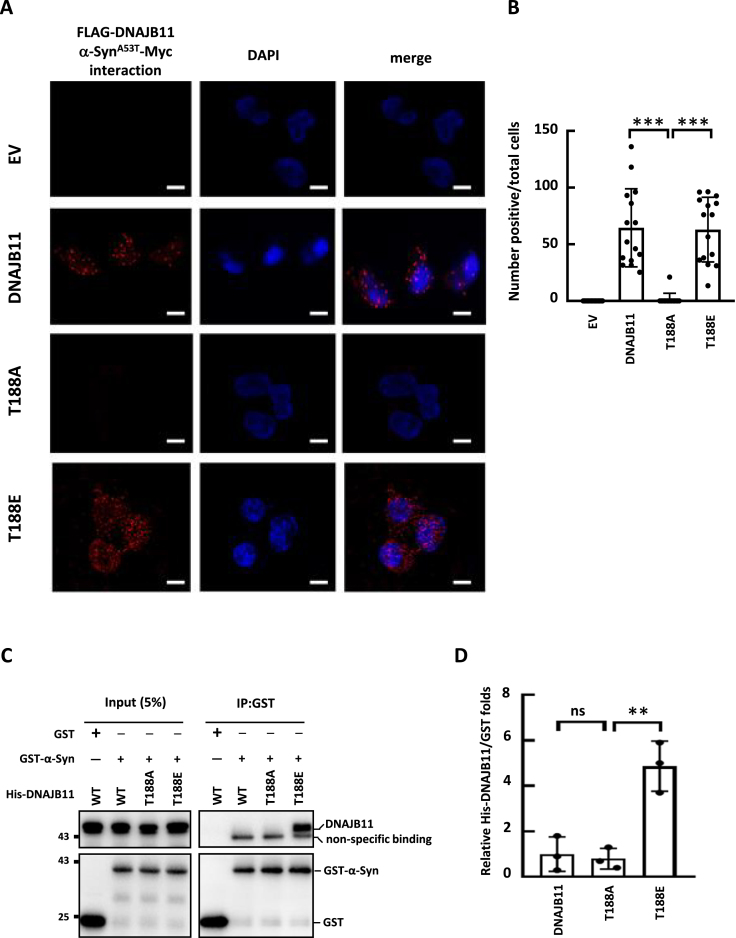
Phosphorylated DNAJB11 interacts with α-synuclein in neuronal cells and *in vitro*. (**A**) SH-SY5Y cells transiently expressing FLAG-tagged WT or mutant DNAJB11 along with Myc-taggedα-synuclein^A53T^ were treated with etoposide for 48-h and then Duolink PLA between FLAG-DNAJB11 and α-synuclein^A53T^-Myc was performed. Empty vector (EV) was used as a negative control. DNA was stained with DAPI. Each red spot represents a single interaction. (**B**) Quantification of the number of proximity ligation spots per cell was determined by counting cells in five randomly chosen fields using the ImageJ software. (**C**) Recombinant GST and GST-α-synuclein *E. coli* lysates were mixed with His6-DNAJB11 WT or mutants *E. coli* lysate and glutathione agarose was added *in vitro*. Samples were bound, washed, eluted, and resolved by SDS-PAGE. Western blotting was conducted and detected with GST and DNAJB11 antibodies. (**D**) Quantification of the relative fold of DNAJB11 was performed using ImageJ (Student's *t*-test, **P < 0.01, ***P < 0.001).

To further examine the interaction between DNAJB11 and α-synuclein in neuronal cells, we performed a proximity ligation assay (PLA) under etoposide treatment. SH-SY5Y cells were treated with etoposide, followed by PLA. The PLA images showed that a fluorescent signal was not observed with the empty vector or DNAJB11 T188A ectopic expression (Figure 6A and B). In contrast, when the PLA assay was conducted in the presence of WT DNAJB11 or T188E (Figure 6A and B), readily detectable red fluorescence appeared, signifying an association between DNAJB11 WT or T188E and α-synuclein^A53T^ proteins. These findings indicate an interaction between T188 phosphorylated DNAJB11 and α-synuclein in SH-SY5Y cells.

To elucidate whether the interaction between DNAJB11 and α-synuclein is direct or facilitated by other factors, recombinant GST-α-synuclein and His6-DNAJB11 were expressed in *E. coli*. The recombinant His6-DNAJB11 WT protein from *E. coli* should not contain T188 phosphorylation. GST-α-synuclein was immobilized on glutathione-Sepharose beads to co-purify His6-tagged DNAJB11. The GST affinity purification assay results revealed that the DNAJB11 T188E mutant was co-purified with GST-tagged α-synuclein. In contrast, GST-tagged α-synuclein did not co-purify DNAJB11 WT and T188A (Figure 6C and D). These findings suggest a direct binding between phosphorylated DNAJB11 and α-synuclein.

We also delineated the potential structure of DNAJB11 using AlphaFold2 (Supplementary Figure S4, bottom panel). The T188 residue is prominently positioned on the surface of the Cys-rich domain II (depicted in red) in the substrate binding domain, potentially rendering it accessible to its client. This suggests the possibility of its impact on the interaction between DNAJB11 and α-synuclein.

### DNAJB11 T188 phosphorylation promotes the outgrowth of neurites

**Figure 7.**
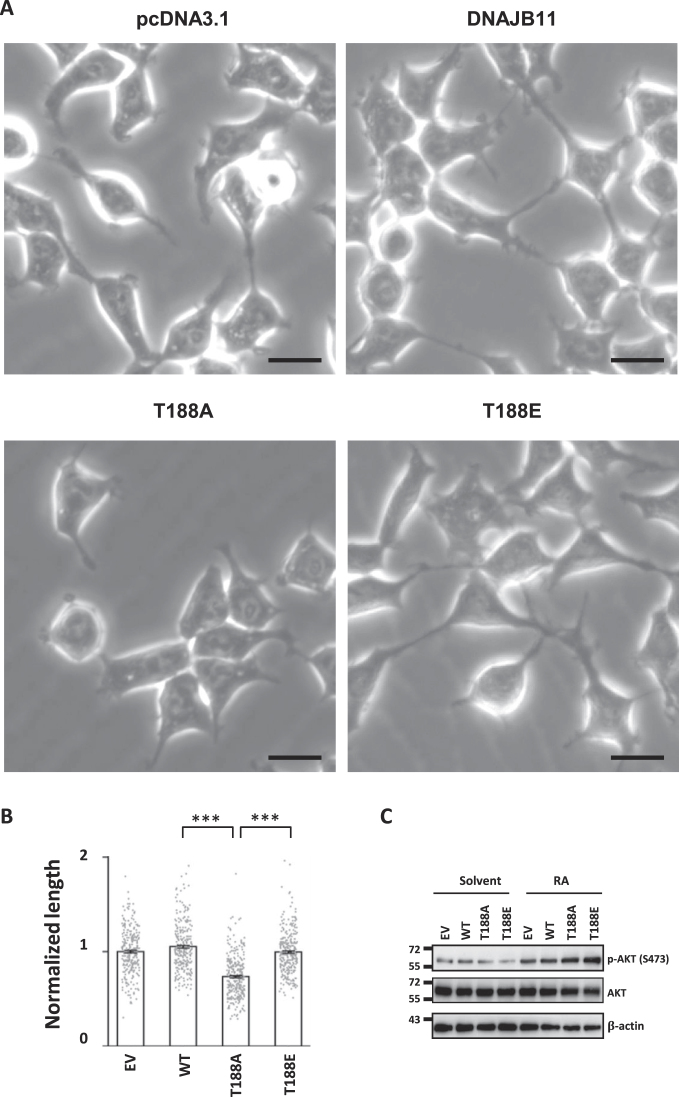
The DNAJB11 T188A mutation reduces neurite length in RA-treated cells. (**A**) DNAJB11 WT, T188A, and T188E expressing SH-SY5Y cells were sequentially incubated with 10 μM RA in a culture medium with 2.5% of FBS for 7 days. Light microscopic images show SH-SY5Y neurite morphology (Zeiss, Axio Observer 7). Scale bar, 100 μm. (**B**) Quantification of neurite outgrowth was analyzed (Student's *t*-test, ***P < 0.001). (**C**) The levels of p-AKT (S473) and AKT protein expression were assessed using Western blotting.

Neurite outgrowth is crucial for processes such as neuronal migration, synapse formation, and the establishment of neural circuits, all of which are essential for the proper functioning of the nervous system (68). To investigate whether the ectopic expression of DNAJB11 WT and mutants affects neuronal function, we examined the neurite outgrowth of SH-SY5Y cells expressing DNAJB11 WT or T188 mutants. Retinoic acid (RA) was used to induce neuronal cell differentiation for neurite outgrowth (69). Compared to the DNAJB11 WT cells, neurite length was significantly reduced in the T188A mutant-expressed cells but recovered in T188E-expressed cells (Figure 7A and B), indicating that the regulation of DNAJB11 T188 phosphorylation is critical for neurite morphogenesis. Additionally, we observed phosphorylation of AKT at Ser-473 (Figure 7C), a phosphorylation indicative of the rapid increase induced by RA in neural differentiation of SH-SY5Y neuroblastoma cells (70).

### Phosphorylation of DNAJB11 is elevated in *SNCA* p.A53T mice and the brains of individuals with PD

**Figure 8.**
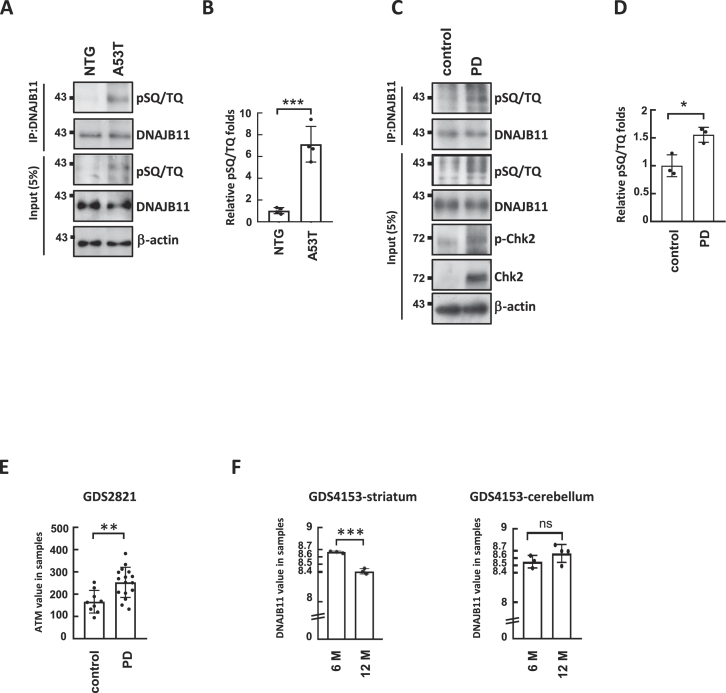
DNAJB11 T188 phosphorylation is increased in *SNCA* p.A53T mice and PD patients. (**A**) Lysates from *SNCA* p.A53T mice substantia nigra sections were subjected to IP analysis by an anti-DNAJB11 antibody. Immunoprecipitates were probed with anti-pSQ/TQ antibodies. Five percent of lysates used for IP were loaded as input and probed with anti-DNAJB11 and pSQ/TQ antibodies. The pSQ/TQ levels were assessed in the brain lysates derived from the midbrain substantia nigra sections obtained from both non-transgenic (NTG) mice (n = 4) and A53T mice (n = 4) at 8 months of age. β-actin was used as a loading control. (**C**) Lysates were prepared from human normal control and PD brains. IP analysis was performed with an anti-DNAJB11 antibody. Immunoprecipitates were probed with anti-pSQ/TQ antibodies. Five percent of lysates used for IP were loaded as input and probed with anti-DNAJB11, anti-p-CHK2, anti-CHK2, and pSQ/TQ antibodies. β-actin is used as a loading control. (**B**, **D**) The amounts of pSQ/TQ and DNAJB11 were quantified, using the Image J software. **(E, F)** The gene expression levels of ATM (**E**) and DNAJB11 (**F**) in the substantia nigra of PD patients and various age groups of mice using GEO profiles (GDS2821 and GDS4153). The results of the quantitative analysis are shown as the relative values to the NTG or normal controls, respectively (Student's *t*-test, *P < 0.05, **P < 0.01, ***P < 0.001).

To detect the DNAJB11 phosphorylation *in vivo*, we investigated the phosphorylation of DNAJB11 in a mouse model of PD. These mice express a missense mutant form of the human α-synuclein (*SNCA*) gene, specifically the p.A53T variant, under the control of the murine prion promoter (37). Western blotting of immunoprecipitated DNAJB11 revealed an elevated phosphorylation of DNAJB11 in transgenic *SNCA* p.A53T PD mice (Figure 8A and B), in comparison to littermate non-transgenic (NTG) control mice.

In response to DNA breaks, the activation of ATM can result in the phosphorylation of numerous substrates. This process serves to facilitate both efficient and accurate DNA repair. Alternatively, ATM activation can trigger a transcriptional program, culminating in cell-cycle arrest in G1, senescence, or apoptosis (71,72). Mutations in various genes responsible for encoding crucial proteins in DNA repair have been demonstrated to play a role in the advancement of PD (73). To explore the connection between DNAJB11 phosphorylation and PD progression, we conducted a DNAJB11 IP assay using human brain lysates from both healthy individuals and PD patients. The detection of CHK2 phosphorylation suggested the potential activation of the ATM pathway in PD cells (Figure 8C). Amazingly, we observed an elevated level of DNAJB11 phosphorylation in PD brain lysates compared to those from healthy controls (Figure 8C and D), indicating an increased activity of DNAJB11-mediated α-synuclein folding in individuals with PD.

Additionally, we assessed the gene expression levels of ATM and DNAJB11 in the substantia nigra of PD patients (39) and various age groups of mice using GEO profiles (40,41). In comparison to the control group, the expression level of ATM is up-regulated in the substantia nigra brain lysates of PD patients (Figure 8E). Moreover, the gene expression levels of DNAJB11 are down-regulated in the striatum, but not in the cerebellum, in older mice at 21 months (Figure 8F). These findings suggest that ATM-mediated DNAJB11 phosphorylation may interact with α-synuclein, potentially reducing α-synuclein aggregation and influencing the progression of PD.

## Discussion

DNAJB11, a member of JDPs, collaborates with the HSP70 chaperone BIP to mitigate the accumulation of misfolded proteins, thereby contributing to cellular proteostasis (34,53,54). While its crucial role in cellular protein folding and quality control has been reported, the detailed regulation remained unclear. Here we unravel a potential stress response between genomic instability and PD pathology. Under DNA DSBs, α-synuclein aggregation increases and DNAJB11 undergoes phosphorylation by the ATM kinase to alleviate the α-synuclein aggregation. DNAJB11 T188 phosphorylation specifically mitigates α-synuclein aggregation, a protein linked to neurodegenerative diseases. While DNAJB11 reduces α-synuclein aggregation, the other two ER-stress-induced JDPs, DNAJB9 and DNAJC10, do not provide the same function, underscoring the client selection and specificity in the diverse chaperone-co-chaperone system. Co-IP assay and PLA demonstrate the interaction between DNAJB11 and α-synuclein. Furthermore, DNAJB11 T188 phosphorylation influences neurite outgrowth, a critical process for nervous function. These findings illuminate the complicated but distinct role of DNAJB11 in cellular stress response and α-synuclein-specific proteostasis, providing valuable insights into its implications for PD and shedding light on the molecular interactions governing its function.

Upon DNA DSBs, the DSB repair mechanism is orchestrated by the phosphorylation of various proteins, encompassing DNA repair factors, cell cycle regulators, and chromatin remodeling factors, under the positive influence of ATM (71,72). Sustained activation of ATM signaling has been observed in murine models of PD (15,19). Our research, establishing DNAJB11 as a newfound ATM substrate, not only serves as an illustrative instance of ATM signaling mediating the JDP-HSP70 regulatory pathway but also unveils that the HSP70-related co-chaperone can undergo phosphorylation by ATM, contributing to DNA damage response to cope with the folding stress and suggesting a crosstalk between two hallmarks of aging (74). The observation that overexpression of DNAJB11 T188A mutation induces α-synuclein aggregation may suggest a dominant-negative effect of the T188A substitution. While losing the ability to recruit its client α-synuclein, DNAJB11 T188A may still associate with HSP70 BIP. This association may prevent these BIP from the delivery of the endogenous client-binding JDPS, thereby impeding the folding cycle of BIP.

A hallmark of PD is the pathological accumulation of misfolded α-synuclein clumps in nerve cells (1). The elimination of these abnormal aggregates necessitates effective degradation mechanisms, including chaperone-mediated autophagy, macroautophagy, and ubiquitin-proteasome pathways (75). A previous study demonstrated that DNAJB6 can mitigate α-synuclein-induced pathology in an animal model of PD (76). Despite these insights, the precise involvement of JDPs in the processes of α-synuclein aggregation and clearance remains elusive. Here, we unveil the significant role of DNAJB11 in α-synuclein aggregation. Both DNAJB6 and DNAJB11 are members of the JDPs and function as molecular co-chaperones dedicated to HSP70-mediated protein quality control and the prevention of misfolded protein aggregation. However, the precise distinction between DNAJB6 and DNAJB11 in the clearance of α-synuclein aggregates needs further elucidation. The subcellular localization of these co-chaperones may also influence their functions. Although both DNAJB6-HSPA8 and DNAJB11-BIP systems play roles in protein folding and quality control, the intricacies of their collaboration or competition in the context of α-synuclein clearance may remain an active area of research. Further investigations are warranted to comprehensively understand their respective contributions in this context.

Mutations in many genes (such as *SNCA*, *LRRK2*, *PRKN*, *DJ1*, *PINK1* and *ATP13A2*) have conclusively been shown to cause familial parkinsonism (77). However, it can only explain less than ten percent of the susceptibility factors for PD (78), implying that epigenetic alteration and/or posttranslational modifications may be a key reason for disease formation. Amino acid substitutions and/or modifications can have notable impacts on protein-protein interactions, particularly when modifications occur at binding interfaces or active site clefts. Such substitutions have the potential to obstruct access to the active site, modify recognition, alter specificity, or influence binding affinity. Our structural analysis suggests that the phosphorylation changes the charge of T188 within the DNAJB11 which may result in adjustments to its surface properties. Given that charge is a fundamental determinant of protein structure and function, modifications in amino acid charge could impact the protein's interactions with other molecules, such as the client protein α-synuclein, and potentially affect the client recruitment in the folding processes. Understanding these structural alterations stemming from changes in amino acid charge is pivotal for deciphering the functional consequences and potential implications for protein homeostasis, particularly concerning molecular co-chaperones like DNAJB11, which play crucial roles in PD-related client folding.

Remarkably, our data indicate an elevation in the phosphorylation of DNAJB11 in *SNCA* p.A53T mice and the brains of individuals with PD. We hypothesize that the accumulation of age-related somatic damage, coupled with a breakdown in compensatory mechanisms, could contribute to an accelerated phosphorylation of DNAJB11 in PD. This phosphorylation may play a crucial role in reducing inappropriate protein aggregation, suggesting a potential link between DNAJB11 phosphorylation dynamics and the pathogenesis of PD.

In summary, our study reveals that ATM-driven phosphorylation of DNAJB11 increases α-synuclein binding, modulates HSP70 function, and prevents α-synuclein aggregation. Consequently, it is plausible that the ATM-driven DNAJB11 T188Q phosphorylation, by influencing the association of clients, plays a significant role in coping with environmental change and controlling the pathogenesis of PD. These findings may carry crucial implications for unraveling the underlying mechanisms of PD and hold promise for the development of novel therapeutic approaches in the future.

## Supplementary Material

ugae007_Supplemental_File

## Data Availability

The data underlying this article are available in the article and in its online supplementary data. Expression analysis of the ATM and DNAJB11 genes utilized data from Gene Expression Omnibus (GEO) accessions GDS2821 (39) and GDS4153 (40,41).
