# Supplementary material for: ATM-mediated co-chaperone DNAJB11 phosphorylation facilitates α-synuclein folding upon DNA double-stranded breaks
Source: NAR Mol Med. 2024 May 13;1(2):ugae007. doi: 10.1093/narmme/ugae007 (PMC12430012; doi:10.1093/narmme/ugae007)
Supplement: ugae007_Supplemental_File [file ugae007_Supplemental_File.pdf]

SUPPLEMENTARY DATA

Supplementary Figure 1

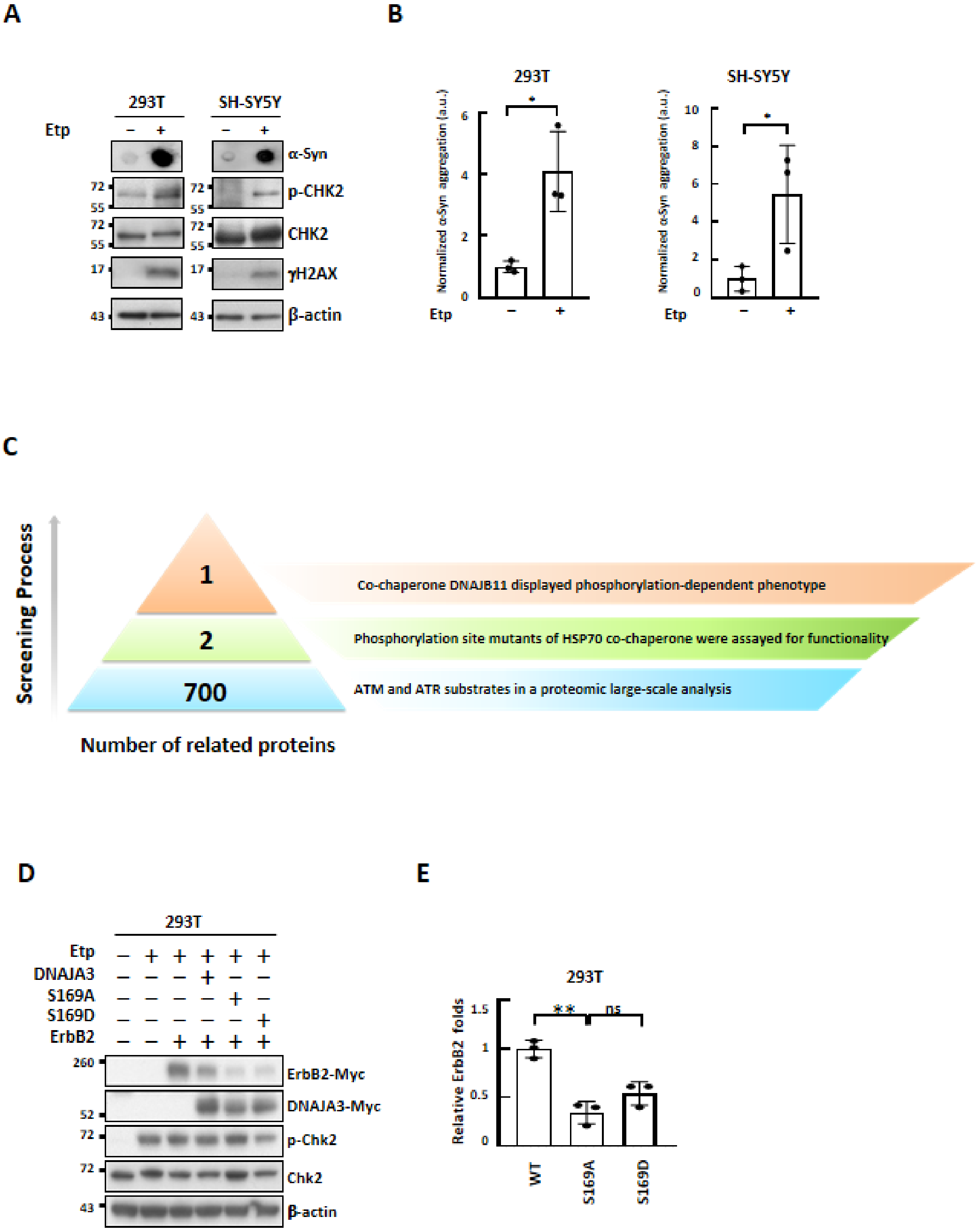

Figure S1. Functional screen for the roles of the HSP70 co-chaperones in neurodegenerative diseases in human neuronal cells.

(A) 293T and SH-SY5Y cells were treated with 10 μM of etoposide or solvent DMSO for 48-h. Etoposide-treated 293T and SH-SY5Y cells displayed increased SDS-insoluble aggregation of α-synuclein. γ-H2AX, the initial step in recruiting DNA repair proteins, serves as a biomarker for DNA DSBs. **(B)** The results were quantified as described in Figure 1. **(C)** A schematic diagram illustrates the steps involved in candidate screening. **(D)** DNAJA3 S169A mutation does not inhibit DNAJA3-induced ErbB2 degradation. 293T cells were transfected with DNAJA3 WT and mutants along with ErbB2, followed by treatment with 5 μM of etoposide for 24-h. Expression levels of ErbB2 were detected by Western blotting. β-actin serves as an internal control. **(E)** The amounts of ErbB2 and DNAJA3 were quantified, using the Image J software. The results of the quantitative analysis are shown as the relative values to the WT (Student's t-test, *, $p < 0.05$, **, $p < 0.01$).

Supplementary Figure 2

```
Human      --MAPQNLSTFCLLLLYLIGAVIAGRDFYKILGVPRSASIKDIKKAYRKLALQLHPDRNP 58
Mouse      --MAPQNLSTFCLLLLYLIGTVIAGRDFYKILGVPRSASIKDIKKAYRKLALQLHPDRNP 58
Zebrafish  MAIRGMKLSSVCFLLLYLILTVFAGRDFYKILGVSRSASVKDIKKAYRKLALQLHPDRNQ 60
Fruit fly  --MRTLAFILVVQIALCLLSVAFAGRDFYKILNVKKSASTNEIKKAYRKLAKELHPDKNK 58
Nematode   --MRILNVSL-LVLASSLVAFVECGRDFYKILGVAKNANANQIKKAYRKLAKELHPDRNQ 57

Human      DDPQAQEKFQDLGAAYEVLSDSEKRKQYDTYGEEGLKDG-HQS-SH-------------- 58
Mouse      DDPQAQEKFQDLGAAYEVLSDSEKRKQYDTYGEEGLKDG-HQS-SH-------------- 102
Zebrafish  DDPNAQDKFADLGAAYEVLSDEEKRKQYDAYGEEGLKEG-HQS-SH-------------- 104
Fruit fly  DDPSASEKFQDLGAAYEVLSDPDKRKTYDRCGEECLKKD-GMMDHG-------------- 103
Nematode   DDEMANEKFQDLSSAYEVLSDKEKRAMYDRHGEEGVAKMGGGGGGG-------------- 103

Human      ----------------GDIFSHFFGDFG---------FMFGGTPRQ-------------- 123
Mouse      ----------------GDIFSHFFGDFG---------FMFGGTPRQ-------------- 123
Zebrafish  ----------------GDIFSSFFGDFG---------FMFGGNRQP-------------- 125
Fruit fly  ----------------ADPFASFFGDFG---------FHFGNG-DP-------------- 123
Nematode   ----------------HDPFSSFFGDFF-----------GGGGGHG-------------- 122

Human      ----------------QDRNIPRGSDIIVDLEVTLEEVYAGNFVEVVRNKPVARQAPGKRK 168
Mouse      ----------------QDRNIPRGSDIIVDLEVTLEEVYAGNFVEVVRNKPVARQAPGKRK 168
Zebrafish  ----------------AGRDIPRGNDIVLDLEVTLEEVYSGNFVEVVRIKPVAKEAPGKRK 170
Fruit fly  ----------------HEHQTARGANIVMNLYVTLEELYSGNFVEIVRNKPVLKPATGTRK 168
Nematode   ----------------GEEGTPKGADVTIDLFVTLEEVYNGHFVEIKRKKAVYKQTSGTRQ 167

Human      CNCRQEMRTTQLGPGRFQMTQEVVCDECPNVKLVNEERTLEVEIEPGVRDGMEYPFIGEG 228
Mouse      CNCRQEMRTTQLGPGRFQMTQEVVCDECPNVKLVNEERTLEVEIEPGVRDGMEYPFIGEG 228
Zebrafish  CNCRQEMRTTQLGPGRFQMTQEVVCDECPNIKLVNEERTLEVEIEQGVRDEMEYPFIGEG 230
Fruit fly  CNCRQEMVTRNLGPGRFQMIQQTVCDECPNVKLVNEERTLEVEVEAGMVDGQETRFVAEG 228
Nematode   CNCRHEMRTEQMGQGRFQMFQVKVCDECPNVKLVQENKVLEVEVEVGADNGHQQIFHGEG 227

Human      EPH-VDGEPGDLRFRIKVVKHPIFERRGDDLYTNVTISLVESLVGFEMDITHLDGHKVHI 287
Mouse      EPH-VDGEPGDLRFRIKVVKHRIFERRGDDLYTNVTVSLVEALVGFEMDITHLDGHKVHI 287
Zebrafish  EPH-IDGEPGDLRFRIKVLKHPVFERRGDDLYTNVTISLVEALVGFEMDITHLDGHKVHI 289
Fruit fly  EPH-LDGEPGDLIIKIMQTPHKSFQRKGDDLYTNVTISLQDALIGFTMNITHLDGHAVSI 287
Nematode   EPH-IEGDPGDLKFKIRIQKHPRFERKGDDLYTNVTISLQDALNGFEMEIQHLDGHIVKV 286

Human      SRDKITRPGAKLWKKGEGLPNFDNNNIKGSLIITFDVDFPKEQLTEEAREGIKQLLKQGS 347
Mouse      SRDKITRPGAKLWKKGEGLPNFDNNNIKGSLIITFDVDFPKEQLTEEAKEGIKQLLKQGP 347
Zebrafish  VRDKITKPGSRIWKKGEGLPSFDNNNIRGSLIITFDVDFPKEQLDDQQKDGIKQLLKQAP 349
Fruit fly  TREKITWPGARIRKKGEGMPNYENNNLQGNLYITFDVEFPKKELTDAEKEDLKKILDQAS 347
Nematode   QRDKVTWPGARLRKKDEGMPSLEDNNKKGMLVVTFDVEFPKTELSDEQKAQIIEILQQNT 346

Human      V-QKVYNGLQGY 358
Mouse      V-QKVYNGLQGY 358
Zebrafish  S-QKVYNGLQGY 360
Fruit fly  I-NRVYNGL--- 355
Nematode   VKPKAYNGL--- 355
```

Figure S2. Sequence alignment of DNAJB11 and its homologs.

The amino acid sequences of DNAJB11 and its predicted homologs were aligned using the Clustal Omega program. The conservation of residues is labeled in gray. The locations of T178Q and T188Q are marked in red.

Supplementary Figure 3

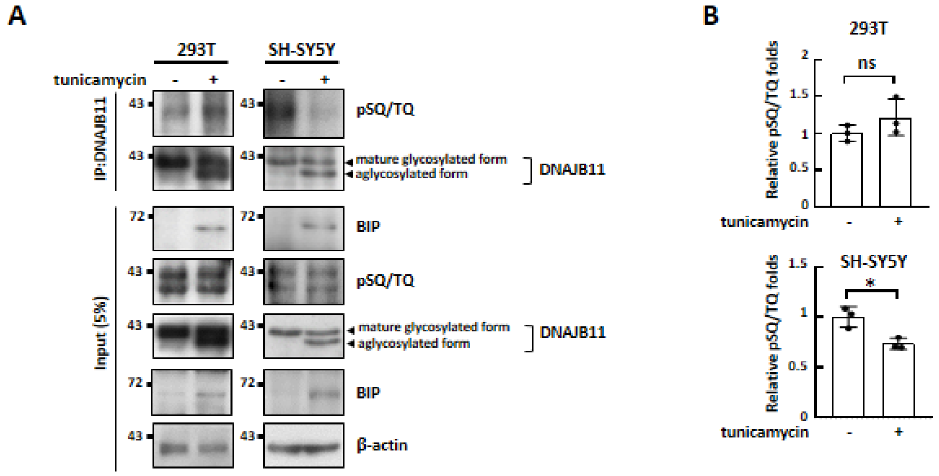

Figure S3. Tunicamycin treatment does not induce DNAJB11 T188 phosphorylation.

(A) IP analysis of DNAJB11 phosphorylation. 293T and SH-SY5Y cells were treated with 10 mg/ml of tunicamycin or solvent for 3-h. IP analysis was performed with an anti-DNAJB11 antibody. Immunoprecipitates were sequentially probed with anti-pSQ/TQ antibodies. Five percent of lysates used for IP were loaded as input and probed with anti-pSQ/TQ, anti-DNAJB11, and anti-BIP antibodies. The upper DNAJB11 band is the mature glycosylated form, while the lower band is the aglycosylated form. β-actin was used as a loading control. **(B)** The amounts of pSQ/TQ and DNAJB11 were quantified, using the Image J software. The results of the quantitative analysis are shown as the relative values to the DMSO control (Student's t-test, *, $p < 0.05$).

Supplementary Figure 4

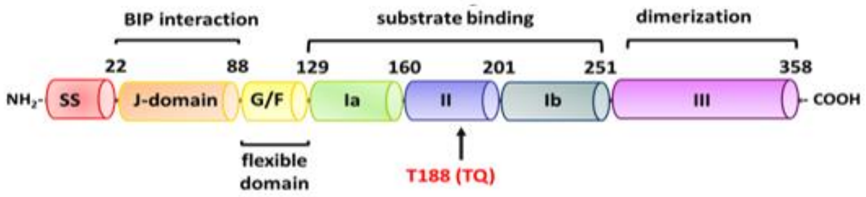

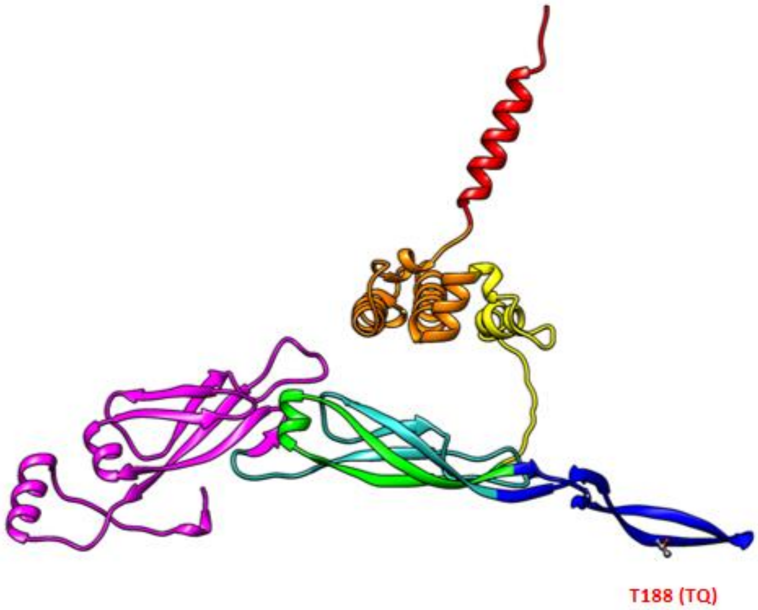

Figure S4. The predicted structure of DNAJB11.

The image presents an overlay of the ribbon structure of DNAJB11, featuring its domain architecture. The upper panel illustrates the structural components of DNAJB11, including the cleavable N-terminal signal sequence (ss), J-domain, flexible Gly/Phe-rich domain (G/F), bifurcated substrate binding domain I (Ia and Ib), Cys-rich domain II, and C-terminal dimerization domain III. The highlighted section denotes the T188Q residue.
